# Reported Prevalence of Inherited Thrombophilia in Pregnancy: Impact of Cohort Selection on Screening Implications—A Systematic Review

**DOI:** 10.3390/diagnostics16152466

**Published:** 2026-08-05

**Authors:** Maria Fulina, Lucian Șerbănescu, Georgeta-Camelia Cozaru, Elena Danteș, Elena Dumea, Eugenia-Alina Radu, Elena Mocanu

**Affiliations:** 1 County Clinical Emergency Hospital “Sf. Ap. Andrei”, 900591 Constanta, Romania; maria.fulina@yahoo.com (M.F.); georgiana.cozaru@365.univ-ovidius.ro (G.-C.C.); 2 Faculty of Medicine, Ovidius University of Constanta, Aleea Universității no.1 , Campus—Building B, 900470 Constanta, Romania; elena.dantes@365.univ-ovidius.ro (E.D.); elenadumea@yahoo.com (E.D.); elena.mocanu@univ-ovidius.ro (E.M.); 3 Center for Research and Development of the Morphological and Genetic Studies of Malignant Pathology (CEDMOG), “Ovidius” University of Constanta, 900591 Constanta, Romania; 4 Academy of Romanian Scientists, 3 Ilfov Street, 050094 Bucharest, Romania; 5 Doctoral School of Medicine, Faculty of Medicine, Ovidius University of Constanta, 1 University Alley, Campus—Corp B, 900470 Constanta, Romania; 6 Clinical Hospital of Pneumopthisiology Constanta, 40 Santinelei Street, 900002 Constanta, Romania; 7 Clinical Hospital of Infectious Diseases, 900178 Constanta, Romania

**Keywords:** inherited thrombophilia, pregnancy, prevalence, recurrent pregnancy loss, screening, Factor V Leiden, prothrombin mutation, obstetric complications

## Abstract

**Background/Objectives**: Inherited thrombophilia (IT) has been extensively investigated as a potential contributor to adverse pregnancy outcomes; however, reported prevalence varies widely across studies. This systematic review aimed to evaluate how cohort selection influences the reported prevalence of inherited thrombophilia in pregnancy and to explore the implications of this variability for clinical interpretation and screening practices. **Methods**: A systematic review was conducted following PRISMA 2020 guidelines. PubMed, Scopus, Web of Science, and Google Scholar were searched. A total of 5480 records were identified, and 7 observational studies (cohort and case–control) met inclusion criteria. Data were synthesized qualitatively due to heterogeneity. **Results**: Prevalence ranged from 0% to 14%, depending on population characteristics. The largest cohort study reported an inherited thrombophilia prevalence of 8.1%, comparable to the general population. Higher rates were observed in referral-based and antiphospholipid Syndrome (APS)-associated cohorts, while case–control studies showed no significant differences between affected individuals and controls. Ethnic variability was also observed. **Conclusions**: These findings should be interpreted as patterns observed across heterogeneous study designs rather than as definitive evidence of causality. Current evidence does not clearly support universal screening in pregnancy; however, the findings should be interpreted cautiously because several included studies were limited by small sample sizes and methodological heterogeneity.

## 1. Introduction

Pregnancy is characterized by a finely regulated shift toward a hypercoagulable state, representing a physiological adaptation aimed at minimizing peripartum hemorrhage [1]. This prothrombotic tendency is mediated by increased levels of clotting factors, reduced fibrinolytic activity, and alterations in natural anticoagulant pathways [1]. While beneficial in normal gestation, this hemostatic imbalance has been implicated in the pathogenesis of thromboembolic events and placental vascular complications when additional procoagulant factors are present [1,2].

Inherited thrombophilia (IT) encompasses a group of genetic conditions associated with an increased tendency toward thrombosis [3]. The most commonly studied abnormalities include Factor V Leiden (FVL) mutation, Prothrombin G20210A mutation, and deficiencies of antithrombin, protein C, and protein S [3,4]. These conditions have been proposed as potential contributors to adverse pregnancy outcomes through mechanisms involving microvascular thrombosis, impaired trophoblast invasion, and reduced uteroplacental perfusion [4]. Such pathophysiological processes have been hypothesized to underlie clinical entities including recurrent pregnancy loss (RPL), intrauterine fetal death, preeclampsia, fetal growth restriction, and placental abruption [5].

Despite strong biological plausibility, the clinical significance of inherited thrombophilia in obstetrics remains a matter of ongoing debate. Over the past decades, numerous studies have attempted to establish a causal relationship between thrombophilic mutations and adverse pregnancy outcomes [5,6,7]. However, the results have been highly inconsistent. While some studies report an increased prevalence of thrombophilia in women with recurrent miscarriage or other complications, others have failed to demonstrate significant associations or have shown prevalence rates comparable to those observed in the general population [8]. Notably, large cohort data indicate that the prevalence of inherited thrombophilia in women with recurrent miscarriage does not exceed background population levels, challenging the assumption of a direct etiological role [8,9].

A critical factor contributing to these discrepancies is the heterogeneity of study populations. Many investigations are conducted in highly selected cohorts, such as patients referred to tertiary care centers, women with multiple pregnancy losses, or individuals with concomitant autoimmune conditions. Such populations are inherently enriched for adverse outcomes, increasing the likelihood of detecting thrombophilic abnormalities [10]. This introduces a substantial risk of selection bias, which may lead to overestimation of prevalence and misinterpretation of clinical significance.

Furthermore, case–control studies designed to evaluate associations between thrombophilia and obstetric complications frequently demonstrate no statistically significant differences between affected individuals and controls. For example, studies assessing prothrombin gene mutations in women with thrombotic obstetric complications have reported similar mutation frequencies in both groups, suggesting that these genetic variants may not represent independent risk factors [11,12]. Similarly, investigations in certain populations have shown complete absence of specific mutations, such as Factor V Leiden, underscoring the influence of ethnic and genetic variability on prevalence estimates [13].

The clinical implications of these uncertainties are substantial. The perception of inherited thrombophilia as a major etiological factor has led to widespread screening practices in obstetric populations, often in the absence of clear evidence of benefit [14]. This approach raises concerns regarding overdiagnosis, unnecessary interventions, and increased healthcare costs. Moreover, anticoagulant therapies initiated on the basis of thrombophilia detection are not without risk and may expose patients to potential complications without demonstrable improvement in pregnancy outcomes [15].

Given these considerations, there is a need for a comprehensive evaluation of the available evidence, with particular emphasis on the relationship between study population characteristics and the reported prevalence of inherited thrombophilia. Understanding the extent to which cohort selection influences prevalence estimates is essential for accurately interpreting the literature and for informing evidence-based clinical practice.

Therefore, the primary aim of this systematic review is to evaluate how cohort selection influences the reported prevalence of inherited thrombophilia in pregnancy across different clinical populations. Secondary considerations include the variability in reported associations with obstetric complications and the potential implications of these findings for current screening practices. Specifically, this review seeks to determine whether the observed prevalence reflects a true increase in disease burden or is primarily driven by selection bias, and to explore how these patterns should inform current approaches to testing for inherited thrombophilia in obstetric practice.

## 2. Materials and Methods

### 2.1. Study Design and Reporting Guidelines

This systematic review was conducted and reported in accordance with the Preferred Reporting Items for Systematic Reviews and Meta-Analyses (PRISMA) 2020 statement. The completed PRISMA 2020 Checklist is provided as Supplementary Materials (Table S1).

#### Protocol Registration

The review protocol was prospectively registered in the International Prospective Register of Systematic Reviews (PROSPERO; CRD420261363273) and is publicly accessible (https://www.crd.york.ac.uk/PROSPERO/view/CRD420261363273) (accessed on 15 May 2026).

### 2.2. Eligibility Criteria

Studies were selected according to predefined inclusion and exclusion criteria.

Inclusion Criteria

• Observational studies (cohort, case–control, cross-sectional)
• Studies involving pregnant women or women with obstetric complications (e.g., recurrent pregnancy loss, APS, bleeding)
• Studies reporting laboratory-confirmed inherited thrombophilia
• Studies providing extractable prevalence data

Exclusion Criteria

• Case reports or small case series (<5 patients, unless rare conditions)
• Reviews, editorials, or conference abstracts without full data
• Studies lacking clear prevalence or frequency data
• Studies focusing exclusively on acquired thrombophilia without genetic components

Studies were grouped for synthesis into:

1. Large cohort studies
2. High-risk referral populations
3. Case–control studies
4. Rare thrombophilia cohorts

### 2.3. Information Sources

A comprehensive search was conducted in the following databases:

• PubMed/MEDLINE
• Scopus
• Web of Science
• Google Scholar

Additionally, reference lists of included articles were manually screened to identify further relevant studies. The last search was conducted on 15 April 2026.

### 2.4. Search Strategy

A comprehensive literature search was conducted in PubMed/MEDLINE, Scopus, Web of Science Core Collection, and Google Scholar from database inception to 15 April 2026. The search combined controlled vocabulary (MeSH terms where applicable) and free-text keywords related to inherited thrombophilia, pregnancy, adverse pregnancy outcomes, and prevalence. Search strategies were adapted to the indexing system of each database. The complete database-specific search strategies are provided in Supplementary Table S2. No language or publication date restrictions were applied.

The search syntax was adapted to the indexing system and technical requirements of Scopus, Web of Science, and Google Scholar. The complete database-specific search strategies are provided in Supplementary Table S2. Reference lists of the included studies and relevant review articles were also manually screened to identify additional eligible publications.

### 2.5. Selection Process

**Figure 1:**
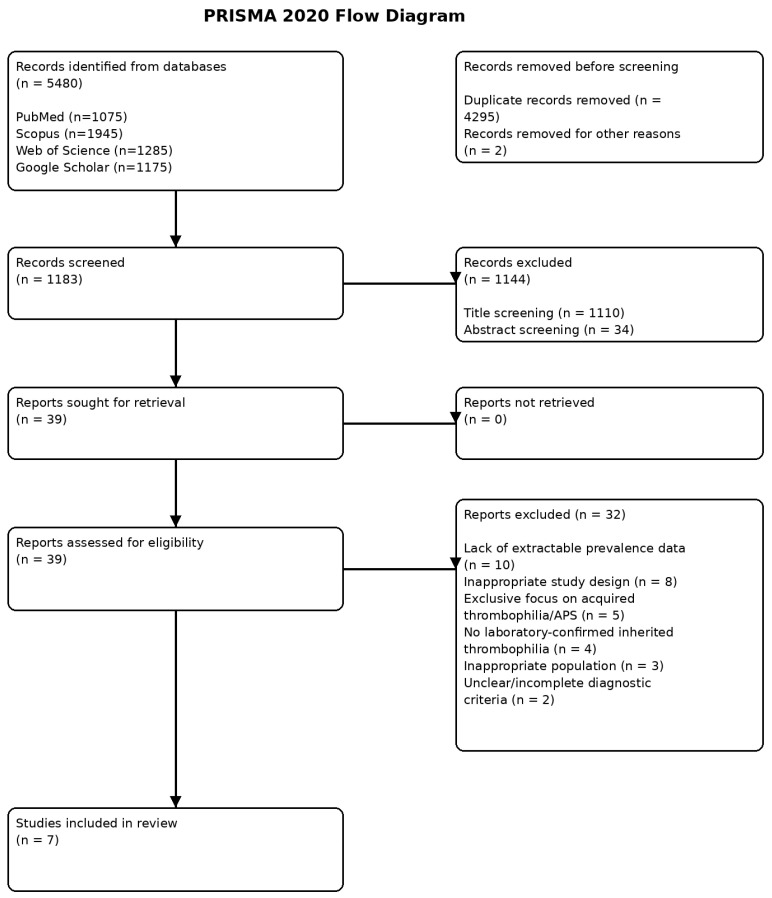
PRISMA 2020 flow diagram for new systematic reviews which included searches of databases and registers only.

Following completion of the database searches, all retrieved records were imported into a reference management software and duplicate records were removed prior to screening. Two reviewers independently screened the titles and abstracts of all remaining records against the predefined eligibility criteria. Full-text articles were subsequently obtained for studies considered potentially eligible. The same reviewers independently assessed the full-text articles for inclusion, and reasons for exclusion were documented. Any disagreements regarding study eligibility were resolved through discussion and consensus. The study selection process is summarized in the PRISMA 2020 flow diagram (Figure 1).

### 2.6. Data Collection Process

Data were extracted independently by two reviewers using a standardized data extraction form developed before data collection. The extracted variables included study design, sample size, study population, type of inherited thrombophilia evaluated, reported prevalence, obstetric outcomes, and the authors’ principal conclusions. Following independent extraction, the reviewers compared the extracted data and resolved discrepancies through discussion to ensure consistency and accuracy.

### 2.7. Data Items

Outcomes

• Primary outcome—prevalence of inherited thrombophilia
• Secondary outcomes—association with obstetric complications and differences between case and control populations

Other Variables

• Study design
• Sample size
• Population characteristics
• Type of thrombophilia (FVL, Prothrombin, MTHFR, AT deficiency)
• Ethnic/geographic background

Assumptions were made when necessary for incomplete reporting, based on study context.

#### Definition of Inherited Thrombophilia

For the purpose of this review, inherited thrombophilia was defined as the presence of one or more of the following well-established genetic defects: Factor V Leiden mutation, prothrombin G20210A mutation, antithrombin deficiency, protein C deficiency, or protein S deficiency.

Antiphospholipid syndromewas not considered an inherited thrombophilia, as it represents an acquired autoimmune condition, and was therefore analysed separately when reported within study populations.

Methylenetetrahydrofolate reductase (MTHFR) polymorphisms were not included in the primary definition of inherited thrombophilia, in line with current evidence and guideline recommendations, which do not support their role as independent risk factors for thrombotic or obstetric complications. When reported in included studies, MTHFR variants were described narratively but were not interpreted as equivalent to established thrombophilic disorders.

### 2.8. Risk of Bias Assessment

The methodological quality of included studies was assessed using the Newcastle–Ottawa Scale (NOS).

Each study was independently evaluated by two reviewers across three domains: selection, comparability and outcome/exposure.

Studies were classified as:

• Low risk (7–9 points)
• Moderate risk (5–6 points)
• High risk (≤4 points)

### 2.9. Effect Measures

The primary effect measure was prevalence (proportion) of inherited thrombophilia.

For comparative studies, differences between groups were interpreted based on reported statistical significance (*p*-values, when available).

### 2.10. Synthesis Methods

Study Inclusion for Synthesis

Studies were included in qualitative synthesis based on:

• Availability of prevalence data
• Comparable population characteristics

Data Preparation

Data were extracted as reported. No imputation or statistical transformation was performed.

Presentation of Results

Results were summarized using:

• Structured tables (Study characteristics, Risk of bias)
• Narrative synthesis

Synthesis Approach

Due to significant heterogeneity in:

• Study populations
• Study design
• Definitions of outcomes

Meta-analysis was not performed. Instead, a qualitative synthesis was conducted.

### 2.11. Exploration of Heterogeneity

Heterogeneity was explored through subgroup comparison based on:

• Population type (RPL, APS, general)
• Study design
• Geographic/ethnic differences

### 2.12. Sensitivity Analysis

Formal sensitivity analysis was not performed due to the absence of quantitative synthesis.

### 2.13. Reporting Bias Assessment

Formal assessment of reporting bias (e.g., funnel plots) was not performed due to the limited number of studies.

### 2.14. Certainty of Evidence

A formal GRADE assessment was planned but not performed due to heterogeneity and limited number of studies. However, overall certainty of evidence was interpreted considering:

• Study design
• Sample size
• Consistency of findings

## 3. Results

### 3.1. Study Selection

The database search identified 5480 records. After removal of duplicates, 1183 records remained for title and abstract screening. Following screening, 1144 records were excluded because they were not related to inherited thrombophilia in pregnancy, did not report pregnancy-related outcomes, or did not provide original clinical data.

A total of 39 full-text articles were assessed for eligibility. Of these, 32 articles were excluded for the following reasons: lack of extractable prevalence data (*n* = 10); inappropriate study design, including narrative reviews, editorials, conference abstracts without full data, or case reports (*n* = 8); exclusive focus on acquired thrombophilia or antiphospholipid syndrome without assessment of inherited thrombophilia (*n* = 5); absence of laboratory-confirmed inherited thrombophilia (*n* = 4); inappropriate or non-pregnancy-related population (*n* = 3); and unclear or incomplete diagnostic criteria (*n* = 2). Finally, 7 studies met the inclusion criteria and were included in the qualitative synthesis.

These exclusion categories were applied at the full-text stage to ensure that only studies reporting laboratory-confirmed inherited thrombophilia with extractable prevalence data in pregnancy-related populations were included.

### 3.2. Study Characteristics

The 7 included studies comprised a total of approximately 1915 participants, with substantial variability in sample size, study design, and clinical context. The main characteristics of the included studies are summarized in Table 1. As shown, there was substantial heterogeneity in study design, population type, and sample size, with studies ranging from large cohort analyses to small case series. Study designs included:

• One large retrospective cohort study (*n* = 1155)
• One medium-sized cohort study in APS patients (*n* = 328)
• One small cross-sectional cohort (*n* = 35)
• Three case–control studies (*n* = 60–257)
• One small case series evaluating rare thrombophilia (*n* = 5)

The populations studied were heterogeneous and included:

• Women with recurrent pregnancy loss (RPL)
• Women with obstetric antiphospholipid syndrome (APS)
• Women with first trimester complications
• General obstetric populations with control groups

The most commonly investigated established inherited thrombophilias were:

• Factor V Leiden (FVL) mutation
• Prothrombin G20210A mutation
• Antithrombin deficiency
• Protein C deficiency (when evaluated)
• Protein S deficiency (when evaluated)

Several studies also reported MTHFR polymorphisms; however, these variants were considered separately because they were not included in the predefined definition of inherited thrombophilia used in this review.

### 3.3. Risk of Bias

Risk of bias was assessed using the Newcastle–Ottawa Scale. The included studies demonstrated variable methodological quality:

• Low risk of bias (*n* = 3): large cohort and well-designed comparative studies with adequate selection and outcome assessment
• Moderate risk of bias (*n* = 3): studies with smaller sample sizes or limited adjustment for confounding factors
• High risk of bias (*n* = 1): small case series (antithrombin deficiency) with limited generalizability

The principal sources of bias included:

• Selection bias due to referral-based populations
• Small sample sizes in several studies
• Lack of standardized diagnostic criteria across studies

The results of the risk of bias assessment are presented in Table 2. Most studies were classified as low to moderate risk of bias, with only one study considered high risk due to small sample size and limited generalizability.

### 3.4. Results of Individual Studies

#### 3.4.1. Shehata et al., 2022 [7] (UK, Retrospective Cohort, *n* = 1155)

This large retrospective cohort study included women with recurrent first-trimester miscarriage managed in two specialist recurrent miscarriage clinics. Inherited and acquired thrombophilia were systematically evaluated, with inherited defects primarily comprising Factor V Leiden and prothrombin G20210A mutations [6].

The prevalence of inherited thrombophilia was 8.1%, and the overall prevalence of any thrombophilia (inherited or acquired) was 9.2%, values comparable to those reported in the general population. These findings suggest no enrichment of inherited thrombophilia in this clinically high-risk but relatively unselected referral cohort.

This study is central to the main objective of the review, as it shows that in a large, rigorously investigated population of women with recurrent miscarriage, the prevalence of inherited thrombophilia remains similar to background rates, rather than being markedly inflated by referral bias [6].

#### 3.4.2. Camacho Sáez et al., 2024 [6] (Spain, Retrospective Cohort, *n* = 328)

This retrospective cohort comprised women with obstetric antiphospholipid syndrome (APS), representing a highly selected population with a strong predisposition to placenta-mediated complications. Alongside antiphospholipid antibodies, several inherited thrombophilic defects were assessed, although the exact testing panel varied across patients [5].

The prevalence of inherited thrombophilia in this APS cohort was approximately 14%, higher than that reported in general obstetric populations and in less selectively enriched cohorts. However, when standard APS treatment was applied, no statistically significant differences in maternal or fetal outcomes were observed between women with and without inherited thrombophilia.

This study [5] illustrates how cohort selection—here, an APS population—can yield higher observed prevalence estimates without demonstrating a corresponding independent effect on clinical outcomes, reinforcing the importance of contextualizing prevalence within the underlying risk profile of the population.

#### 3.4.3. Frikha et al., 2023 [8] (Tunisia, Cross-Sectional Study, *n* = 35)

This cross-sectional study evaluated women with recurrent pregnancy loss, focusing on Factor V Leiden and prothrombin G20210A mutations [7]. The sample was relatively small but clinically homogeneous, consisting of women investigated in a tertiary setting for unexplained pregnancy loss.

The reported prevalence of inherited thrombophilia was 8.6%, broadly consistent with the estimates from larger cohort studies. Thrombophilic mutations were more frequently observed among women with an additional history of thrombotic events, suggesting that their clinical impact may depend on coexisting risk factors rather than on pregnancy loss alone.

Although limited by sample size, this study [7] supports the notion that variation in prevalence may be more pronounced within specific subgroups (for example, those with both recurrent pregnancy loss (RPL) and venous thromboembolism (VTE)) than across the overall population of women with pregnancy loss.

#### 3.4.4. Alam et al., 2018 [5] (Pakistan, Case–Control Study, *n* = 60)

This case–control study examined the prothrombin G20210A mutation in 30 women with thrombotic obstetric complications (included preeclampsia, placental abruption, intrauterine growth restriction, and intrauterine fetal death) and 30 matched controls with uncomplicated pregnancies [4].

The heterozygous prothrombin mutation was identified in 3.3% of cases and in none of the controls, and this difference did not reach statistical significance (*p* = 0.5). The odds of mutation-positive status in cases were therefore low and imprecisely estimated.

These findings indicate that no statistically significant association between the prothrombin G20210A mutation and thrombotic obstetric complications was demonstrated in this study [4]. However, the small sample size limits statistical power, and clinically relevant associations cannot be excluded. Therefore, these results should be interpreted with caution and confirmed in larger prospective studies [9,11].

#### 3.4.5. Stamatian et al., 2006 [4] (Romania, Case–Control Study, *n* = 75)

This case–control study included 36 women with first-trimester bleeding and 39 controls with uncomplicated pregnancies [3]. Factor V Leiden, prothrombin G20210A, and MTHFR polymorphisms were evaluated. Because MTHFR polymorphisms were outside the predefined definition of inherited thrombophilia adopted in this review, they are reported separately from the established thrombophilic defects.

The distribution of inherited thrombophilic mutations was similar between cases and controls, and no statistically significant differences were observed for factor V Leiden or prothrombin G20210A. MTHFR polymorphisms were frequent but showed no clear association with the presence of bleeding.

This study did not demonstrate statistically significant differences in the prevalence of established inherited thrombophilic defects between women with first-trimester bleeding and controls [3]. Nevertheless, because of the relatively small sample size, these findings should not be interpreted as definitive evidence against a possible association in specific patient populations [9,11].

#### 3.4.6. Kobashi et al., 2005 [1] (Japan, Case–Control Study, *n* = 257)

This case–control study investigated genetic thrombophilia in Japanese women with recurrent spontaneous abortion of unexplained etiology and in controls with successful pregnancies [1]. Factor V Leiden mutation and MTHFR C677T polymorphisms were evaluated. While the study reported both markers, only Factor V Leiden contributed to the synthesis of inherited thrombophilia prevalence because isolated MTHFR polymorphisms were not considered established inherited thrombophilic disorders.

No cases of factor V Leiden mutation were identified in either the recurrent miscarriage group or the control group, while MTHFR variants showed no significant association with recurrent pregnancy loss [1].

These findings underscore the role of ethnic and genetic background in shaping the distribution of inherited thrombophilia, demonstrating that some classic defects, such as factor V Leiden, may be essentially absent in certain populations, with important implications for the interpretation of prevalence and for the generalizability of screening strategies.

#### 3.4.7. Ilonczai et al., 2015 [3] (Hungary, Case Series, *n* = 5)

This small case series described pregnancies in five women with hereditary antithrombin deficiency, a rare but high-risk inherited thrombophilia [2]. The report detailed the course of pregnancy under different anticoagulation strategies and the occurrence of maternal venous thromboembolism and adverse fetal outcomes.

The series reported a high incidence of thromboembolic events and pregnancy loss despite prophylaxis in some cases, underscoring the substantial individual risk associated with severe antithrombin deficiency [2].

However, given the very small sample size and the rarity of the condition, these data are not generalisable to the broader obstetric population. Instead, the study primarily illustrates that rare, high-risk thrombophilias may have a major impact at the individual level while contributing minimally to overall population prevalence.

### 3.5. Results of Syntheses

#### 3.5.1. Summary of Contributing Evidence

The included studies varied substantially in both design and risk of bias. Higher-quality studies, particularly large cohort analyses, consistently reported lower and more stable prevalence estimates compared to smaller or referral-based studies.

#### 3.5.2. Synthesis of Prevalence Estimates

Across all studies, the reported prevalence of inherited thrombophilia ranged from:

• 0% in certain populations
• ~3–5% in general or controlled populations
• ~8–10% in recurrent pregnancy loss cohorts
• Up to ~14% in highly selected APS populations

Due to substantial heterogeneity in study design, populations, and diagnostic criteria, meta-analysis was not performed, and a qualitative synthesis was considered more appropriate.

#### 3.5.3. Exploration of Heterogeneity

The primary sources of heterogeneity were:

• Cohort selection (dominant factor)
• Study design (cohort vs case–control)
• Ethnic and geographic variability

For instance, the absence of Factor V Leiden mutation in Japanese populations illustrates the impact of genetic background on prevalence estimates.

#### 3.5.4. Sensitivity Considerations

Although formal sensitivity analysis was not conducted, indirect comparisons suggest that studies with broader, less selective populations consistently reported lower prevalence estimates and higher prevalence estimates were associated with referral-based or highly selected populations.

Across the included studies, MTHFR polymorphisms were reported descriptively when available but were excluded from the primary qualitative interpretation of inherited thrombophilia prevalence because they are not considered clinically established inherited thrombophilic disorders.

### 3.6. Reporting Biases

Formal assessment of reporting bias was not feasible due to the limited number of studies. However, the potential for publication bias should be considered, particularly given the tendency to preferentially publish studies demonstrating positive associations.

### 3.7. Certainty of Evidence

The overall certainty of evidence is considered moderate to low, due to:

• Heterogeneity in study populations and design
• Limited number of large, high-quality studies
• Inconsistent findings across smaller studies

However, the consistency of results in larger cohort studies provides increased confidence in the main conclusions regarding prevalence and lack of association.

## 4. Discussion

### 4.1. Principal Findings

The systematic review explored how cohort selection may influence the reported prevalence of inherited thrombophilia in pregnancy [4,6]. Across seven heterogeneous studies, prevalence estimates varied widely between populations. Large, less selected cohorts showed prevalences close to those expected in the general population. In contrast, referral-based and very high-risk cohorts often reported higher prevalences [5,6]. Case–control studies did not find consistent differences between women with and without obstetric complications [1,3,4]. Overall, the findings suggest that part of the variability in reported prevalence may be explained by differences in cohort definition and baseline risk rather than by a uniform increase in disease burden [3,14,15].

Unlike previous reviews that primarily examined whether inherited thrombophilia is associated with adverse pregnancy outcomes, the present review specifically evaluated how cohort selection influences reported prevalence estimates. By focusing on differences in study populations rather than solely on clinical outcomes, this review provides a methodological perspective that helps explain inconsistencies across the existing literature and supports a more cautious interpretation of prevalence data and screening recommendations [9,11].

### 4.2. Influence of Cohort Selection on Prevalence Estimates

To address the primary objective of this review, the findings can be interpreted according to study type and population, as each contributes differently to understanding how cohort selection influences reported prevalence.

Across the included studies, the reported prevalence of inherited thrombophilia varied according to the characteristics of the study populations. Large cohort studies involving broader obstetric populations generally reported prevalence estimates comparable to those observed in the general population, suggesting that inherited thrombophilia is not substantially overrepresented in unselected women with adverse pregnancy outcomes [6,9]. In contrast, referral-based cohorts and women with obstetric antiphospholipid syndrome consistently demonstrated higher reported prevalence, reflecting the enrichment of high-risk patients and selective referral patterns rather than a true increase in disease frequency [5,7]. These findings emphasize that cohort selection is an important source of heterogeneity across studies and should be considered when interpreting reported prevalence estimates and comparing results between different populations [9,11]. Consequently, prevalence estimates should be interpreted within the clinical context of the populations investigated and should not be generalized to all pregnant women or used alone to justify routine thrombophilia screening [14].

Taken together, these observations are consistent with the idea that differences in reported prevalence across studies are at least partly explained by which populations are tested, in what clinical context, and under which indications, rather than by a simple underlying increase in disease frequency.

### 4.3. Interpretation According to Thrombophilia Type

The interpretation of the findings should consider the specific inherited thrombophilic defect evaluated rather than treating inherited thrombophilia as a single clinical entity. The included studies predominantly investigated Factor V Leiden and the prothrombin G20210A mutation because these represent the most common inherited thrombophilic variants encountered in clinical practice [6,16].

Large cohort studies generally reported prevalence estimates for these common mutations that were comparable to those observed in the background population, whereas smaller case–control studies produced inconsistent findings [6,17,18]. Importantly, several of the comparative studies included relatively small numbers of participants, limiting statistical power. Consequently, the absence of statistically significant differences should not be interpreted as definitive evidence that these variants have no clinically relevant role in selected obstetric populations [3,4,19].

The findings differed for the rarer inherited thrombophilias. Antithrombin deficiency, and to a lesser extent deficiencies of protein C and protein S, are uncommon but are associated with a substantially increased individual risk of venous thromboembolism [2,14]. Their rarity means that they contribute minimally to the overall prevalence of inherited thrombophilia in pregnancy, yet they remain clinically important because identification of affected women may influence counselling, thromboprophylaxis, and pregnancy management [14,20].

Several included studies also reported MTHFR polymorphisms [1,3]. However, these variants were intentionally excluded from the primary definition of inherited thrombophilia used in this review because current evidence and international guideline recommendations do not support their classification as clinically relevant inherited thrombophilic disorders or their routine inclusion in thrombophilia testing [21,22]. They were therefore described only to accurately reflect the findings of the original studies and were not interpreted alongside established inherited thrombophilic defects.

Taken together, these observations suggest that the clinical relevance of inherited thrombophilia depends on the specific genetic defect being considered. Interpreting all inherited thrombophilic abnormalities as a single homogeneous group may obscure important differences in prevalence, thrombotic risk, and potential clinical implications [14,21].

### 4.4. Comparison with Existing Literature and Guidelines

The findings of the present review are consistent with an evolving body of evidence suggesting that the relationship between inherited thrombophilia and adverse pregnancy outcomes is more complex than originally proposed. Earlier studies frequently reported stronger associations; however, many were conducted in highly selected populations or included relatively small sample sizes, increasing the risk of selection bias and limiting the generalizability of their findings [3,4].

More recent observational studies and systematic reviews have reported more modest and less consistent associations, particularly in unselected obstetric populations [6,9]. The present review extends these observations by demonstrating that differences in cohort selection represent an important methodological explanation for the wide variation in reported prevalence estimates. This perspective helps explain why studies investigating similar thrombophilic defects have produced apparently conflicting results [9,11].

Current international guidelines recommend selective rather than universal testing for inherited thrombophilia during pregnancy [21,22]. Testing is generally reserved for women with a personal history of venous thromboembolism, a strong family history of thrombosis, or other high-risk clinical circumstances, rather than being routinely performed in all women with adverse pregnancy outcomes [21,22]. The findings of the present review support this individualized approach because higher reported prevalence was largely observed in clinically selected populations rather than in broader obstetric cohorts [5,6].

Importantly, the absence of consistent statistically significant associations across several included studies should not be interpreted as proof that inherited thrombophilia has no role in adverse pregnancy outcomes. Many of the available studies were limited by relatively small sample sizes, heterogeneous populations, and differences in laboratory testing strategies, reducing their ability to detect modest but clinically meaningful associations [7,9]. Consequently, current evidence supports cautious interpretation of negative findings while emphasizing the need for adequately powered prospective studies using standardized diagnostic criteria [11,21].

### 4.5. Clinical Implications

The findings of the present review have important implications for clinical practice. Routine screening for inherited thrombophilia in all women with adverse pregnancy outcomes is not supported by the available evidence because reported prevalence varies considerably according to cohort selection and the underlying clinical characteristics of the population being investigated [6,9,21].

Instead, decisions regarding thrombophilia testing should be individualized and guided by the overall clinical context. Women with a personal history of venous thromboembolism, a strong family history of inherited thrombophilia or thrombosis, or other well-recognized high-risk clinical situations are more likely to benefit from targeted laboratory evaluation than women undergoing routine investigation after isolated obstetric complications [21,22].

The present findings also highlight the importance of interpreting positive thrombophilia test results cautiously. Detection of an inherited thrombophilic defect should be considered alongside the patient’s personal history, family history, obstetric characteristics, and other thrombotic risk factors rather than being regarded as an isolated explanation for adverse pregnancy outcomes [14,21].

From a research perspective, future studies should recruit clearly defined populations, apply standardized diagnostic criteria, and distinguish between unselected obstetric populations and clinically enriched referral cohorts. Such methodological improvements would facilitate more reliable comparisons between studies and help identify patient subgroups in whom inherited thrombophilia testing may provide meaningful clinical benefit [9,11].

Future research should focus on large, multicenter prospective cohort studies enrolling women during the preconception period or early pregnancy using standardized inclusion criteria and uniform laboratory testing protocols for established inherited thrombophilias [20]. Participants should be stratified according to baseline thrombotic risk, including a personal history of venous thromboembolism, a family history of thrombosis, and previous adverse pregnancy outcomes, to allow evaluation of inherited thrombophilia within clinically relevant risk groups [21]. Obstetric outcomes, including recurrent pregnancy loss, preeclampsia, fetal growth restriction, placental abruption, stillbirth, and venous thromboembolism, should be prospectively recorded using standardized diagnostic definitions to improve comparability across studies [11]. Such a study design would minimize selection bias, reduce methodological heterogeneity, and provide more robust evidence regarding the independent contribution of inherited thrombophilia to adverse pregnancy outcomes, while helping to identify the women most likely to benefit from targeted testing and preventive interventions [9].

## 5. Conclusions

This systematic review demonstrates that reported prevalence estimates of inherited thrombophilia in pregnancy vary substantially across studies and are strongly influenced by cohort selection, baseline clinical risk, and population characteristics [5,6,9]. This methodological heterogeneity represents an important source of variability and should be considered when interpreting the existing literature and comparing prevalence estimates between studies [9,11].

The available evidence did not consistently demonstrate statistically significant associations between inherited thrombophilia and adverse pregnancy outcomes across unselected obstetric populations [6,9]. However, several included studies were limited by relatively small sample sizes, heterogeneous study populations, and differences in diagnostic strategies, meaning that clinically relevant associations cannot be excluded in specific high-risk patient groups [3,4,7].

Overall, the findings of this review support current guideline recommendations favoring selective rather than universal thrombophilia testing during pregnancy. Decisions regarding testing should be based on individual clinical risk factors, including a personal history of venous thromboembolism, a strong family history of thrombosis, and other well-established indications, rather than on obstetric complications alone.

Future multicenter prospective cohort studies using standardized diagnostic criteria, uniform laboratory testing protocols, and clearly defined obstetric outcomes are needed to determine the independent contribution of inherited thrombophilia to adverse pregnancy outcomes and to identify women who are most likely to benefit from targeted thrombophilia testing and evidence-based preventive management [21].

## 6. Limitations

The findings of this review should be interpreted in the context of several important constraints. The number of included studies was limited, and the available evidence was heterogeneous in terms of study design, population characteristics, and thrombophilia testing strategies. In addition, one large cohort contributed a substantial proportion of the total sample, which may influence the overall interpretation of prevalence patterns.

A considerable proportion of the included studies were conducted in referral-based or otherwise highly selected populations, which limits the generalizability of the findings to broader obstetric populations. Differences in diagnostic criteria, laboratory methods, and definitions of clinical outcomes further complicate direct comparison across studies.

Finally, the absence of quantitative synthesis precludes the estimation of pooled prevalence and limits the ability to formally assess statistical heterogeneity or publication bias. These factors should be considered when interpreting the conclusions and highlight the need for larger, well-designed studies with standardized methodologies.

## Figures and Tables

**Table 1:** Characteristics of included studies.

Author (Year)	Country	Study Design	Sample Size	Genetic Markers Assessed	Main Outcome	Key Findings
Shehata et al., 2022 [7].	UK	Retrospective cohort	1155	FVL, Prothrombin, acquired thrombophilia	Prevalence of thrombophilia	IT prevalence 8.1%, comparable to general population;
Camacho Sáez et al., 2024 [6].	Spain	Retrospective cohort	328	Inherited thrombophilia (various)	Maternal and fetal outcomes	IT prevalence ~14%; no worse outcomes under treatment
Frikha et al., 2023 [8].	Tunisia	Cross-sectional	35	FVL, Prothrombin mutation	Prevalence of mutations	IT prevalence 8.6%; associated with thrombotic history
Alam et al., 2018 [5].	Pakistan	Case–control	60	Prothrombin G20210A	Association with complications	Mutation 3.3% in cases vs 0% controls; not significant
Stamatian et al., 2006 [4].	Romania	Case–control	75	FVL, Prothrombin, MTHFR	Association with early complications	No significant difference between groups
Kobashi et al., 2005 [1].	Japan	Case–control	257	MTHFR, FVL	Genetic association	No FVL mutation detected; no association
Ilonczai et al., 2015 [3].	Hungary	Case series	5	Antithrombin deficiency	Pregnancy outcomes	High complication rate; rare condition

**Table 2:** Risk of Bias Assessment of Included Studies.

Study	Selection (0–4)	Comparability (0–2)	Outcome/Exposure (0–3)	Total Score (0–9)	Risk Level
Shehata et al., 2022 [7].	4	2	3	9	Low
Camacho Sáez et al., 2024 [6].	4	2	3	9	Low
Frikha et al., 2023 [8].	3	1	2	6	Moderate
Alam et al., 2018 [5].	3	1	2	6	Moderate
Stamatian et al., 2006 [4].	3	1	2	6	Moderate
Kobashi et al., 2005 [1].	4	2	2	8	Low
Ilonczai et al., 2015 [3].	2	0	1	3	High

## Data Availability

The data presented in this study are available on request from the corresponding author. No new data were created or analyzed in this study.

## Supplementary Materials

The following supporting information can be downloaded at: https://www.mdpi.com/article/10.3390/diagnostics16152466/s1. Table S1. PRISMA 2020 checklist. Table S2. Database-specific search strategies used for the systematic review.

## References

[1] Kobashi G., Kato E.H., Morikawa M., Shimada S., Ohta K., Fujimoto S., Minakami H., Yamada H. (2005). MTHFR C677T polymorphism and factor V Leiden mutation are not associated with recurrent spontaneous abortion of unexplained etiology in Japanese women. Semin. Thromb. Hemost..

[2] Middeldorp S., Nieuwlaat R., Baumann Kreuziger L., Coppens M., Houghton D., James A.H., Lang E., Moll S., Myers T., Bhatt M. (2023). American Society of Hematology 2023 guidelines for management of venous thromboembolism: Thrombophilia testing. Blood Adv..

[3] Ilonczai P., Oláh Z., Selmeczi A., Kerényi A., Bereczky Z., Pókadi R. (2015). Management and outcome of pregnancies in women with antithrombin deficiency: A single-center experience and review of literature. Blood Coagul. Fibrinolysis.

[4] Stamatian F., Caracostea G., Mureșan D., Bartok I., Militaru M., Procopciuc L. (2009). The evaluation of inherited thrombophilic conditions in patients with bleeding in the first trimester of pregnancy. HVM Bioflux.

[5] Alam M.A., Ayyub M., Ahmad S.Q., Ali N. (2018). Prothrombin G20210A gene mutation in pregnant females with thrombotic obstetric complications. Pak. Armed Forces Med. J..

[6] Camacho Sáez B., Martínez-Taboada V.M., Merino A., Comins-Boo A., González-Mesones B., Del Barrio-Longarela S. (2024). Impact of inherited thrombophilia in women with obstetric antiphospholipid syndrome: A single-center study and literature review. Biomedicines.

[7] Shehata H., Ali A., Silva-Edge M., Haroon S., Elfituri A., Viswanatha R. (2022). Thrombophilia screening in women with recurrent first trimester miscarriage: Is it time to stop testing?. BMJ Open.

[8] Frikha R., Turki F., Abdelmoula N., Rebai T. (2023). Maternal inherited thrombophilia and recurrent pregnancy loss: A Tunisian study and review of literature. Afr. Health Sci..

[9] Turesheva A., Aimagambetova G., Ukybassova T., Kaldygulova L., Azizan A. (2023). Recurrent pregnancy loss: Etiology, risk factors, and management. Int. J. Women’s Health.

[10] Dicks A.B., Moussallem E., Stanbro M., Walls J., Gandhi S., Gray B.H. (2024). A comprehensive review of risk factors and prevention of venous thromboembolism in pregnancy. J. Clin. Med..

[11] Padrnos L., Gangaraju R. (2024). Inherited thrombophilia and recurrent miscarriage: Is there a role for anticoagulation?. Hematology.

[12] Kirovakov Z., Hinkova N., Konova E. (2024). The impact of inherited thrombophilia on first trimester pregnancy outcomes. Arch. Med. Biomed. Res..

[13] Neagu M., Patrascu N., Rosca I. (2024). Management of high-risk pregnancy associated with inherited thrombophilia. J. Obstet. Gynecol..

[14] Merz L.E., Bassa B., Ní Áinle F., Fogerty A.E. (2025). Thrombotic complications in pregnancy: Current evidence and management strategies. Clin. Obstet. Gynecol..

[15] Maglic D., Mandic-Markovic V., Mikovic Z., Maglic R., Anicic R., Mandic M. (2025). Impact of inherited thrombophilia on pregnancy outcomes and effect of LMWH therapy. Medicina.

[16] Leibenhaut K.C., Tinkle M.B. (2026). Factor V Leiden and venous thromboembolism during pregnancy and postpartum. Nurs. Women’s Health.

[17] Badulescu O.-V., Hancianu M., Mircea C., Bojan A., Tesoi D.-F., Vladeanu M.C., Ciocoiu M., Frasinariu O.-E., Plesoianu C.E., Iliescu-Halitchi D. (2026). A pilot study on multigenic thrombophilic risk in recurrent pregnancy loss: Interactions between MTHFR polymorphisms and classical thrombophilia-associated SNPs. Int. J. Mol. Sci..

[18] Chen Y., Wang T., Liu X., Ye C., Xing D., Wu R., Li F., Chen L. (2022). Low molecular weight heparin and pregnancy outcomes in women with inherited thrombophilia: A systematic review and meta-analysis. J. Obstet. Gynaecol. Res..

[19] Quenby S., Booth K., Hiller L., Coomarasamy A., de Jong P.G., Hamulyák E.N., Scheres L.J., van Haaps T.F., Ewington L., Tewary S. (2023). Heparin for women with recurrent miscarriage and inherited thrombophilia (ALIFE2): An international open-label randomized controlled trial. Lancet.

[20] Liu X., Chen Y., Ye C., Xing D., Wu R., Li F., Chen L., Wang T. (2021). Hereditary thrombophilia and recurrent pregnancy loss: A systematic review and meta-analysis. Hum. Reprod..

[21] Grandone E., Tiscia G.L., Mastroianno M., Larciprete G., Kovac M., Tamborini Permunian E., Lojacono A., Barcellona D., Bitsadze V., Khizroeva J. (2021). Findings from a multicentre observational study on reproductive outcomes in women with unexplained recurrent pregnancy loss: The OTTILIA registry. Hum. Reprod..

[22] American College of Obstetricians and Gynecologists (2018). Inherited thrombophilias in pregnancy: ACOG Practice Bulletin No. 197. Obstet. Gynecol..

