# Supplementary material for: Reported Prevalence of Inherited Thrombophilia in Pregnancy: Impact of Cohort Selection on Screening Implications—A Systematic Review
Source: Diagnostics (Basel). 2026 Aug 5;16(15):2466. doi: 10.3390/diagnostics16152466 (PMC13464732; doi:10.3390/diagnostics16152466)
Supplement: Supplementary file 1 [file diagnostics-16-02466-s001.zip › diagnostics-4329880-Table S2.pdf]

Supplementary Table S2. Database-specific search strategies used for the systematic review.

Database	Complete search strategy	Search date	Filters applied
PubMed/MEDLINE	("Thrombophilia"[MeSH Terms] OR thrombophilia OR "inherited thrombophilia" OR "Factor V Leiden" OR "prothrombin G20210A" OR "prothrombin mutation" OR "antithrombin deficiency" OR "protein C deficiency" OR "protein S deficiency") AND ("Pregnancy"[MeSH Terms] OR pregnancy OR miscarriage OR "recurrent pregnancy loss" OR "recurrent miscarriage" OR preeclampsia OR "fetal growth restriction" OR "placental abruption" OR "obstetric complications") AND (prevalence OR frequency OR screening)	15 April 2026	No language restrictions; no publication date restrictions
Scopus	TITLE-ABS-KEY (thrombophilia OR "inherited thrombophilia" OR "Factor V Leiden" OR "prothrombin G20210A" OR "prothrombin mutation" OR "antithrombin deficiency" OR "protein C deficiency" OR "protein S deficiency") AND TITLE-ABS-KEY (pregnancy OR miscarriage OR "recurrent pregnancy loss" OR "recurrent miscarriage" OR preeclampsia OR "fetal growth restriction" OR "placental abruption" OR "obstetric complications") AND TITLE-ABS-KEY (prevalence OR frequency OR screening)	15 April 2026	None
Web of Science Core Collection	TS=(thrombophilia OR "inherited thrombophilia" OR "Factor V Leiden" OR "prothrombin G20210A" OR "prothrombin mutation" OR "antithrombin deficiency" OR "protein C deficiency" OR "protein S deficiency") AND TS=(pregnancy OR miscarriage OR "recurrent pregnancy loss" OR "recurrent miscarriage" OR preeclampsia OR "fetal growth restriction" OR "placental abruption" OR "obstetric complications") AND TS=(prevalence OR frequency OR screening)	15 April 2026	None
Google Scholar	"Inherited thrombophilia" AND pregnancy AND prevalence OR "recurrent pregnancy loss" OR miscarriage OR preeclampsia	15 April 2026	Results were ranked by relevance, and the first 200 records were screened; no publication date restriction

Abbreviations: MeSH, Medical Subject Headings; TS, Topic Search; TITLE-ABS-KEY, Title, Abstract, and Keywords.

Search strategies were adapted to the syntax and indexing requirements of each database. Controlled vocabulary (MeSH terms) was used where available and combined with free-text keywords. No language or publication date restrictions were applied. Reference lists of all eligible studies and relevant review articles were manually screened to identify additional studies. Duplicate records were removed before title and abstract screening.
